# ThermoTRP Channels in Nociceptors: Taking a Lead from Capsaicin Receptor TRPV1

**DOI:** 10.2174/157015908783769680

**Published:** 2008-03

**Authors:** Sravan Mandadi, Basil D. Roufogalis

**Affiliations:** 1 Hotchkiss Brain Institute, 3330 Hospital Drive NW, Calgary, Alberta T2N 4N1, Canada;; 2 Faculty of Pharmacy, University of Sydney, NSW 2006, Australia

**Keywords:** Transient receptor potential (TRP), ThermoTRP, TRPV, TRPM, TRPA, nociceptor, pain, phosphorylation, analgesia.

## Abstract

Nociceptors with peripheral and central projections express temperature sensitive transient receptor potential (TRP) ion channels, also called thermoTRP’s. Chemosensitivity of thermoTRP’s to certain natural compounds eliciting pain or exhibiting thermal properties has proven to be a good tool in characterizing these receptors. Capsaicin, a pungent chemical in hot peppers, has assisted in the cloning of the first thermoTRP, TRPV1. This discovery initiated the search for other receptors encoding the response to a wide range of temperatures encountered by the body. Of these, TRPV1 and TRPV2 encode unique modalities of thermal pain when exposed to noxious heat. The ability of TRPA1 to encode noxious cold is presently being debated. The role of TRPV1 in peripheral inflammatory pain and central sensitization during chronic pain is well known. In addition to endogenous agonists, a wide variety of chemical agonists and antagonists have been discovered to activate and inhibit TRPV1. Efforts are underway to determine conditions under which agonist-mediated desensitization of TRPV1 or inhibition by antagonists can produce analgesia. Also, identification of specific second messenger molecules that regulate phosphorylation of TRPV1 has been the focus of intense research, to exploit a broader approach to pain treatment. The search for a role of TRPV2 in pain remains dormant due to the lack of suitable experimental models. However, progress into TRPA1’s role in pain has received much attention recently. Another thermoTRP, TRPM8, encoding for the cool sensation and also expressed in nociceptors, has recently been shown to reduce pain *via* a central mechanism, thus opening a novel strategy for achieving analgesia. The role of other thermoTRP’s (TRPV3 and TRPV4) encoding for detection of warm temperatures and expressed in nociceptors cannot be excluded. This review will discuss current knowledge on the role of nociceptor thermoTRPs in pain and therapy and describes the activator and inhibitor molecules known to interact with them and modulate their activity.

## INTRODUCTION

Pain is an unpleasant experience resulting from complex and interactive series of mechanisms at multiple levels of the nervous system. The afferent pain pathway relays pain signals from the periphery to the brain *via* the spinal cord by a class of nerve fibers called “nociceptors” [181]. Nociceptors (C and Aδ) have peripheral and central terminals originating from cell bodies housed in dorsal root ganglia (DRG). Peripheral terminals innervate skin and viscera, while the central terminals innervate the dorsal horn of the spinal cord. Pain perception or nociception is an integration of the modulatory events that take place in the periphery (site of initial pain), in the dorsal horn (DH) of the spinal cord (primary processing centers), supraspinal relay centers in brain such as the thalamus (secondary processing centers) and the corticolimbic structures. Acute and sub chronic pain serves a physiological function of warning and withdrawal from harmful or noxious stimuli. On the other hand, persistent chronic pain associated with inflammatory tissue damage and or nerve injury is considered pathological. Pathological pain can prolong pain sensation and become maladaptive if left unmanaged or untreated. Also, in pathological pain there is heightened sensitization of nociceptors due to changes in the milieu that regulates sensory transducers to function towards more damaging pain. 

A solution to effectively treat pain originating from such tissue or nerve damage is to better understand the mechanisms of nociceptive transmission of potential sensory transducers of pain and their regulation within the nociceptors. One such major family of sensory transducers in nociceptors belongs to the Transient Receptor Potential (TRP) family of cation channels [139, 34]. The uniqueness of these receptors is that they render the nociceptors polymodal, responding to chemical, thermal and mechanical stimuli. Their unique response to temperature has given them the name thermoTRP’s. These include members from the subfamily vanilloidTRPV (TRPV1, 2, 3 and 4), melastatinTRPM (TRPM8), and ankyrin transmembrane proteins TRPA (TRPA1) [45]. Between them, response to noxious heat is mediated by TRPV1 and TRPV2, innocuous warm temperature by TRPV3 and TRPV4, innocuous cool temperature by TRPM8 and noxious cold by TRPA1 [45]. Discovery of thermoTRP’s as molecular targets for some of the naturally occurring compounds that elicit thermal or painful behavior underlies the basis for such sensory functions of nociceptors. Much of the past, current and future thermoTRP research is based on leads obtained from TRPV1, the first cloned thermoTRP member. In order to achieve significant analgesia from a state of acute or chronic pain following noxious chemical or thermal stimuli and tissue damage to nociceptors it is imperative to target a range of thermoTRP’s for developing new therapeutic strategies.

Several lines of evidence ranging from *in vitro* and *in vivo* studies in animals to humans have proved TRPV1 to be a potential target in nociceptors for the treatment of pathological pain, ranging from inflammation to neuropathies. The paradigm that TRPV1 can serve as a target for alleviating certain pain modalities has generated interest in expanding the search for other thermoTRP’s which will also serve as targets for pain relief. This review will focus on current research scenarios highlighting the role of thermoTRP’s in nociception, with TRPV1 still the front runner in this search. Here we discuss selected thermoTRP’s in the sequence TRPV1, TRPV2, TRPA1, TRPM8, TRPV3 and lastly TRPV4 The selected thermoTRP's represent sensitivity to a range of temperatures from noxious heat (TRPV1, TRPV2) and cold (TRPA1) to innocuous cool (TRPM8) and warmth (TRPV3, TRPV4).

## TRPV1

A new horizon in pain research was realized in 1997 when Julius and colleagues [25] identified the specific receptor responding to the hot chilli pepper active ingredient, capsaicin, in subsets of nociceptors. The name vanilloid receptor 1 (VR1) coined by the group is now re-named TRPV1 under the unified nomenclature for the family of TRP cation channels [34,139]. TRPV1 stands out as the first thermally gated cation channel to be discovered in nociceptors. TRPV1 knockout studies gave clear evidence of the importance of TRPV1 in thermal hyperalgesia, bladder function, reduced fever response and more [23, 24]. Since its cloning, TRPV1 has emerged as an important transducer in several settings of pain and beyond, an update of which is highlighted in the following sections.

### Expression, Physiology and Pathology 

The expression pattern of TRPV1 has been widely studied and there now is a comprehensive amount of data available to define not only its localization but also functional expression in physiology and disease. Functional expression of TRPV1 among sensory neurons includes somatosensory ganglia, namely dorsal root ganglia (DRG), trigeminal ganglia and jugular ganglia. TRPV1 is also expressed in nodose ganglia [146]. While trigeminal ganglia peripheral terminals innervate the face and mouth, the DRG projects its peripheral terminals to the rest of the body. Jugular ganglia receive the glossopharyngeal nerve which innervates the pharynx and tongue. Nodose ganglia receive the vagus nerve whose peripheral terminals innervate viscera of the thorax and abdomen. The majority of these afferents belong to the C and Aδ class of nociceptors. Peripheral TRPV1 positive terminals are located in layers of skin epidermis, gastrointestinal tract (GI), urinary bladder, airways, cardiovasculature and oral cavity [146]. In the spinal cord, while the lamina I of DH is innervated by a peptidergic subset of TRPV1 positive terminals, lamina II is innervated by non-peptidergic TRPV1 terminals. In the brain TRPV1 terminals are located in the solitary tract nucleus and trigeminal nucleus, which receive afferent signals *via* vagal, glossopharyngeal and facial nerves. Other areas of the brain with TRPV1 expression have been reported. They include the ventral medulla, periaqueductyl grey, dorsal raphe nucleus, locus coeruleus, hypothalamus, thalamus, ventral tegmental area, substantia nigra, hippocampus, cerebellum and somatosensory cortex [193]. However, the physiological function of TRPV1 in these areas is still in its infancy with respect to making major claims.

The non-neuronal distribution of functional TRPV1 includes epithelial cells of the GI, airway and bladder; epidermal keratinocytes from human skin; enterocytes; liver; vascular endothelium; mast cells; smooth muscle; fibroblasts; and peripheral mononuclear blood cells. Despite such a wide distribution pattern, nociceptors most abundantly express TRPV1, being in the order of more than 30 times that in other tissues [25]. Such abundance in nociceptors confers to TRPV1 a primary physiological role in transducing pain upon its activation by noxious chemical or thermal stimuli from the external environment. It also confers a role in mediating pathological pain signals resulting from the changing expression and or sensitivity of the receptor to the external or internal environment during disease. 

One component of TRPV1-mediated neuronal dysfunctional states of pain originates at peripheral terminals of nociceptors innervating skin and viscera. These include conditions like neurogenic and non-neurogenic inflammation (thermal hyperalgesia, hyperesthesia and allodynia), neuropathy (trigeminal neuralgia, post-herpetic neuralgia, diabetic neuropathy and nerve injury), cancer pain (mastalgia and bone sarcomas), inflammatory joint pain (osteoarthritis), cardiac pain ( heart pain, cardial ischemia), bladder diseases (hyperreflexia, interstitial colitis and detrusor overreactivity), GI diseases (inflammatory bowel, Crohn’s, ulcerative colitis and gastro-oesophageal reflux), vulvodynia, lung diseases (chronic cough and particulate matter-induced apoptosis), headache (cluster headache and migraine) [37, 75, 205-207].

The other component of TRPV1 mediated pain includes central sensitization at the spinal level, where nociceptors terminate in the superficial DH. Intradermal injection of capsaicin results in primary hyperalgesia to heat and mechanical stimuli in the vicinity of the injection site [113, 188, 189]. This is followed by the development of secondary mechanical hyperalgesia and allodynia in an area surrounding the site [113, 216]. Pain due to secondary hyperalgesia and allodynia involve sensitization of nociceptive terminals in the dorsal horn. Capsaicin stimulates nitric oxide production *via* ill-defined mechanisms, which, in turn, initiates the release of glutamate from terminals of vanilloid-sensitive nociceptors in dorsal horn [177]. Glutamate activates NMDA receptors (NMDAR) on neurons of the dorsal horn, including spinothalamic tract cells. During capsaicin-induced hyperalgesia, there are enhanced responses (sensitization) to glutamate activation of NMDAR [51, 53]. The positive feedback by glutamate on vanilloid-sensitive nerve endings in dorsal horn facilitates the release of SP [120]. Dorsal horn neurons involved in pain transmission express receptors (NK-1Rs) for SP, which is upregulated during inflammatory hyperalgesia [129, 179]. NK-1R antagonists prevent the sensitization of spinothalamic tract neurons after intradermal capsaicin injection [52]. Therefore, NMDAR- and NKR-mediated mechanisms facilitate central sensitization of dorsal horn during development of capsaicin-induced hyperalgesia. However, mechanisms for TRPV1-mediated thermal hyperalgesia during neuropathic pain could not be confirmed, as there was increased TRPV1 expression in uninjured neurons [171]. Also, tactile allodynia prevails in a neuropathic pain model where C nociceptors are ablated by capsaicin, largely due to recruitment of *de novo* TRPV1-positive Aβ afferents for pain signalling following central sensitization [171]. The role of NMDAR in central sensitization during peripheral hypersensitivity-mediated visceral pain involves a TRPV1-mediated component in parallel to mechanisms described for peripheral thermal-hyperalgesia [234]. However, a supraspinal regulation of this condition is also in place, whereby NMDAR activation in the rostral ventro-medial medulla maintains the central sensitization at the spinal cord *via* its descending modulation. Visceral pain is also regulated by other supra-spinal areas, like the cortex and hypothalamus, with TRPV1-positive neurons. These areas control visceral afferent nociceptive processing during diseases associated with emotional states like stress and anxiety [193]. A direct or regulatory role for TRPV1 in such disease states needs further investigation.

In addition to the importance of receptor distribution, two other basic rules for heightened TRPV1-mediated pain processing by the nociceptors can be sensitization and upregulation of expression during disease. An increase in TRPV1 expression occurs in primary sensory neurons after peripheral inflammation and requires retrograde transport of nerve growth factor (NGF). NGF pathways of increased TRPV1 expression include activation of p38 mitogen-activated protein kinase (MAPK) and phosphoionositide 3 kinase (PI3K) and phospholipase C (PLC) [18, 30, 93, 136, 194, 242, 244]. Moreover, protein kinase C (PKC) activation induces rapid delivery of TRPV1 channels to the cell membrane, contributing to the sensitizing effect of this kinase on TRPV1 [142]. Increases in the trafficking of TRPV1 to the periphery contribute to inflammatory pain hypersensitivity [93], a problem that can be easily targeted *via* therapeutic blocking by TRPV1 antagonists. It is the TRPV1 sensitization by a myriad of endogenous activators and modulators that has drawn a great deal of attention, aimed at finding a comprehensive approach to silencing the receptor during specific modalities [170]. Another aspect of TRPV1 is the paradoxical state of desensitization following its activation by agonists, whereby the desensitized TRPV1 represents analgesia. Thus, while newly developed antagonists present a promising avenue to block TRPV1-mediated pain, the age old formula of TRPV1 desensitization by its agonists has not lost its importance. The following sections will address these topics. 

### Activation and Regulation

#### Endogenous Activators

A wide variety of endogenous substances that can activate TRPV1 have been discovered. These include lipids such as *N*-arachidonoyldopamine (NADA), oleoylethanolamide (OEA) and *N*-oleoyldopamine (NODA) [2, 28, 82, 219] and their metabolite anandamide [246], which is also a cannabinoid receptor ligand [44]. 

Other lipid mediators include metabolites of the lipoxygenase (LOXs) pathway, namely, 12- and 15-(*S*)-hydroperoxyeicosatetraenoic acids (12- and 15-HPETE), 5- and 15-(*S*)-hydroxyeicosatetraenoic acids (5- and 15-HETE); leukotriene B4 [85, 183] polyamines (spermine). These endogenous ligands contribute to TRPV1-mediated pain in disease where the receptor is sensitized *via* a variety of signaling cascades, as described in the next section. 

#### Sensitization via Signaling Molecules

Prostaglandin E_2_ (PGE_2_), bradykinin, NGF, extracellular ATP, glutamate, trypsin, prolactin, prokineticin2 (Bv8) are pro-inflammatory mediators that activate their respective receptors EP prostaglandin, B_1_/B_2_ bradykinin, high-affinity TrkA NGF receptor, P2Y_2_, mGlu 5, PAR-2, PRLR and PKR2 G protein-coupled receptors [48, 96, 148, 221]. Activation of these receptors in nociceptors leads to activation of phospholipase C (PLC), phospholipase A_2_ (PLA_2_), adenylyl cyclase, protein kinase C (PKC) and protein kinase A (PKA) signaling pathways. These pathways result in sensitizing TRPV1 indirectly *via* phosphorylation [96]. PLC hydrolyzes phosphatidylinositol 4,5-bisphosphate (PIP_2_) and hydrolysis of PIP_2_ also releases inositol 1,4,5-trisphosphate (IP_3_) and diacylglycerol (DAG), which activate PKC, leading to TRPV1 phosphorylation [83]. Proposed relief of TRPV1 from PIP2 mediated tonic inhibition [30] is debatable as PIP2 was shown to sensitize TRPV1 when applied intracellularly [173, 194]. Other pathways that potentiate TRPV1 are activation by PI3K [244] either *via* calmodulin-dependent kinase II (CaMKII), PKC, or ERK [244] or PKA [185]. The PLA_2_ pathway produces arachidonic acid (AA), which can be converted to 12-hydroperoxyeicosatetraenoic acid (12-HPETE) by 12-lipoxygenase (LOX). HPETE activates TRPV1 [183]. Inhibition of PLC, PKC, PLA_2_, and PKA reduce peripheral nociception [61, 79]. Sensitization of TRPV1 *via* a multitude of signaling cascades during disease poses a challenge to therapy with agonists, while antagonists would prove more beneficial. Pros and cons of potential agonists and antagonists in therapy are discussed in sections below.

#### Mechanisms of Desensitization- the Paradox with Activation

TRPV1 can be desensitized following its activation and desensitization is calcium and phosphorylation-state dependent [212]. Prolonged or repeated application of capsaicin induces a desensitization of TRPV1, representing analgesia, a paradox in pain biology. The calcium dependence of TRPV1 desensitization was reproduced in a non-neuronal context, where desensitization of TRPV1 expressed in Xenopus oocytes required the presence of extracellular calcium [25].Capsaicin-induced desensitization is a complex process with varying kinetic components. A fast component appears to be dependent on intracellular calcium, voltage, and calcineurin activity, while a slower component appears at least to be ATP dependent [49, 110, 167, 215]. Further complexity is overlaid by interactions between factors such as voltage-dependent calcium influx and calcium-dependent phosphatase activity [151, 138, 163]. Recently, advances have been made at the molecular and biochemical level to understand how phosphorylation by protein kinases regulates TRPV1 desensitization.

The cAMP-dependent PKA signal pathway decreases desensitization of TRPV1 wild type. Disruption of phosphorylation at potential PKA phosphorylationsite S116D (replacing serine (S) residue with alanine (A)) [16, 137] prevented desensitization. Unlike PKA-dependent reversal of TRPV1 tachyphylaxis by short repeated applications of capsaicin, acute desensitization of wild type (WT) TRPV1 evokedby a prolonged capsaicin application remained unaffected by PKA.

Mutation of a single amino acid in transmembrane domain 6 (TM6) of TRPV1, Y671K or Y671R (replace tyrosine (Y) with lysine (K) or arginine (R)), dramatically altered the high relative Ca^2+^ permeability and desensitization properties of the receptor [137]. Both mutations Y671K and Y671R showed a decrease in relative permeability for Ca^2+^ over Na^+^ ions and the mutated receptor did not desensitize at all. 

Interestingly, calcium entry following capsaicin application is found to form a CaM/Ca^2+^ complex with a 35-aa segment of TRPV1 and cause desensitization [154]. This was confirmed by disrupting of a 35-aa segmentin TRPV1, which inhibited capsaicin-induced tachyphylaxis and acute desensitization [154].

Reversal of TRPV1 desensitization as a positive feedback-loop for regaining activity was shown to be mediated by CaMKII or PKC [97, 127, 128]. Mutation of TRPV1 at the CaMKII consensus sites of TRPV1 phosphorylation S502 or T704 showed lack of agonist binding. Recovery of the sensitivity of desensitized TRPV1 was achieved *via* PKCε mediated phosphorylation at S800 residue [128].

Current knowledge points to the conclusion that phosphorylated TRPV1 is active and sensitized, while its de-phosphorylated state represents desensitization. Phosphorylation of TRPV1 by kinases appears to be critical for its sensitization, and dephosphorylation by calcineurin appears to be critical for its desensitization. However, further work is still needed to identify the site of de-phosphorylation that determines inactivation of TRPV1. This will make available the molecular determinant that can overcome the influence of the milieu of modulators that can sensitize TRPV1 *via* phosphorylation in disease. These models can be applied to specific disease states that can alter the milieu of relevant second messenger systems. 

### Therapeutic Potential- Agonists Versus Antagonists

This section describes compounds that have been confirmed as TRPV1 agonists or antagonists following the cloning of the receptor, in addition to traditional use of some in pain therapy. Other pharmacological effects in addition to TRPV1-mediated mechanisms are not described here. However, some compounds acting as agonists or antagonists for other thermoTRP’s are included.

#### Vanilloids

TRPV1 had derived its maiden name Vanilloid Receptor subtype 1 (VR1) [25] from the fact that it was cloned with the help of capsaicin (*trans*-8-methyl-*N*-vanillyl-6-nonenamide), which belongs to the vanilloid class of compounds composed of the vanillyl moiety in their chemical structure. Capsaicin to date has been shown to selectively activate TRPV1, thus making it one of the most prolifically used specific pharmacological tools in pain research. Much earlier to the cloning of TRPV1, the hallmark agonist capsaicin has been traditionally in use for pain relief of peripheral origin in different disease settings like chemical or thermal hyperalgesia in neurogenic inflammation, herpes zoster, neuropathy, paresthetica, thoracotomy, mastectomy, amputation, and skin cancer [37, 64, 75, 206, 209]. Other disease states of visceral origin that have found capsaicin useful are bladder detrusor instability, hyperreflexia and migraine. Resiniferatoxin, a phorbol ester with the vanillyl moiety, is an ultrapotent agonist of TRPV1 and has also been under intense clinical trial evaluation for relieving incontinence [38, 187]. Alkaloid piperine (piperinoyl-piperidine), the pungent ingredient of black or white pepper, reduces intestinal motility *in vivo* in mice by a mechanism that seems to involve capsaicin-sensitive neurons [91]. Eugenol, a phenol with vanillyl moiety is derived from clove oil and cinnamon leaf oil [59] and used for toothache, pulpitis, and dentin hyperalgesia [157, 158]. However, eugenol is a nonselective TRPV1 agonist as it is also activates other thermoTRP’s, namely TRPA1 and TRPM8 [11]. The other class of phenol compounds with vanillyl moiety that are derived from ginger include gingerols ([8]-gingerol and [6]-gingerol) used in traditional Chinese medicine for headaches, nausea, colds, arthritis, rheumatological disorders and muscular discomfort [43, 175]. Gingerols also activate TRPA1 [11]. In addition to gingerols, [6]-shogaol [59] is also used for its analgesic properties. Other less effective compounds that are TRPV1 agonists include zingerone, a phenolic ketone metabolite of gingerols, and Capsiate (4-hydroxy-3-methoxybenzyl (*E*)-8-methyl-6-nonenoate) obtained from a non-pungent cultivar of red peppers (as *C. annuum* or *C. frutescens*), named CH-19 Sweet [88, 104]. Typical routes of administration for vanilloids include topical, visceral instillations, injections to epidural or subarachnoid space in the case of deep tissue pain, perineural route in neurogenic inflammation. Such treatment regimens mainly include reversible and or irreversible loss of capsaicin-sensitive C-fibers as a mechanism for analgesic effect. Pungency and irritation of vanilloid compounds have been the major drawbacks in pain therapy. However, synthetic analogs of some of the naturally occurring vanilloids have been developed to overcome the pungency factor, such as NGX-4010 (NeurogesX), which is in phase III trials for postherpetic neuropathy, HIV-associated sensory neuropathy; WL-1002 (Winston Laboratories) is under clinical trial for cluster headache, migraine and osteoarthritic pain; compound 4975 (Anesiva) is under clinical trial for neuropathic and musculoskeletal pain.

#### Non-vanillyl Compounds 

The list of TRPV1 agonists has increased several fold in recent years, to include non-vanillyl naturally occurring agents, some of which are partial antagonists such as the Ginseng derivatives ginsenosides [21]; Cannabidiol, a cannabinoid [133]; Evodia compounds (evodiamine and rutaecarpine), alkaloids from *Evodia rutaecarpa* fruits [78, 106-109, 164]; unsaturated 1,4-dialdehyde terpenes [196]; triprenyl phenol (scutigeral), from *Albatrellus ovinus *[74, 208]; jellyfish and cnidarian envenomations [41]; spider toxins [95] and polygodial and drimanial, unsaturated 1,4-dialdehyde sesquiterpenes isolated from the bark of *Drymis winteri* [9]. However, additional studies are necessary to confirm the precise nociceptive or anti-nociceptive mechanism/s through which some of these compounds interact or modulate the TRPV1 channel. 

#### TRPV1 Antagonists

Since agonists are able to activate nociceptors and cause pain, a paradox that makes their selection for some pain therapies a difficult choice, specific antagonists of TRPV1 have been developed, with the aim to overcome the initial pain factor associated with using agonists. Capsazepine, a first generation TRPV1 antagonist, has been shown to be less promising than expected due to its poor pharmacokinetics [218]. In addition to this capsazepine has shown non-selective blockade of other ion channels such as voltage-sensitive calcium channels [153], nicotinic acetylcholine receptors [121] and TRPM8 [14]. Several pharmaceutical companies like GlaxoSmithKline, Novartis, Astra Zeneca, Amgen, Johnson & Johnson, Neurogen/Merck, Renovis/Pfizer and so on have launched attempts to develop second generation TRPV1 antagonists and some of their concerted efforts are beginning to bear fruit as clinical trials are in progress. A number of these have been developed, including iodo-RTX, SB705498, SB366791, BCTC, NGD-8243, AMG-517, AMG-9810, A-425619, KJM429, JYL1421, JNJ17203212, NGX-4010, WL-1001, WL-1002, A-4975, and GRC-6127. Some of these have reached Phase I and or Phase II clinical trials. 

SB-705498 (GlaxoSmithKline) [169] successfully completed Phase I clinical trials, whereby it reduced capsaicin-evoked flare and heat-evoked pain in non-sensitized skin. UVB-evoked inflammation was also reduced by SB705498. Presently, Phase II trials for the acute treatment of dental pain and migraine is underway (ClinicalTrials.gov identifier: NCT00269022). A-425619 (Abbott) [1-isoquinolin-5-yl-3-(4-trifluoromethyl-benzyl)-urea] inhibited mechanical allodynia induced by skin incision and reversed mechanical hyperalgesia in rat models of chronic and neuropathic pain [56, 77]. AMG-9810 (Amgen) [(*E*)-3-(4-*t*-Butylphenyl)-*N*-(2, 3-dihydrobenzo[*b*] and dioxin-6-yl) acrylamide] reverted thermal and mechanical hyperalgesia induced by complete Freund's adjuvant (CFA) [66]. BCTC [*N*-(4-tertiarybutylphenyl)-4-(3-cholorphyridin-2-yl)tetrahydropryazine-1(2H)-carbox-amide] and Nrgn (Neurogen) significantly attenuated thermal and mechanical hyperalgesia in the chronic constriction injury (CCI) model of neuropathic pain and CFA model of inflammatory pain in rats [111]. GRC 6127 (Glenmark), an orally active antagonist, can reverse both CFA-induced and partial sciatic-nerve legation-induced mechanical hyperalgesia in the rat [111]. JNJ-17203212 (Johnson&Johnson) relieved osteolytic sarcoma-related bone pain in the mouse [205]. Similar to capsazepine and iodo-RTX, JNJ-17203212 can block citric-acid-induced cough in guinea pigs [205]. Other recent literature reports on TRPV1 antagonists include 2-(4-pyridin-2-ylpiperazin-1-yl)-1*H*-benzo[*d*]imidazole com-pound 46ad[156]; 6-aryl-7-isopropylquinazolinones [40]; 5,6-fused heteroaromatic urea A-425619.0 [55]; 4-aminoquinazoline [243]; halogenated thiourea compounds 23c and31b [98]; *N*-tetrahydroquinolinyl, *N*-quinolinyl and *N*-iso-quinolinyl carboxamides [233]; pentacyclic triterpene and oleanolic acid [125].

Despite these promising developments, TRPV1 antagonists are beset with problems of side-effects, largely arising from interference with the physiological function of TRPV1-expressing cells. Recent evidence has shown that orally active TRPV1 antagonists can induce gastric ulcer formation, hypertension, hyperthermia and central nervous system effects [76, 207]. It remains to be seen in clinical trials whether or not the TRPV1 antagonists have favorable therapeutic actions. Some patients on TRPV1 antagonists for pain might be at risk of the possible masking of ischemic pain of cardiac origin, as C-fibers innervating the heart are blocked [162]. Thus TRPV1-ligand effects can be unpredictable in patients with complex cardiovascular problems. At present, it is unclear to what degree these findings apply to humans. Also, TRPV1 antagonists which cross the blood brain barrier may cause CNS side effects.

In addition to the use of agonists or antagonists, substances able to modulate TRPV1 (such as at phosphorylation sites) or to decrease the production of endogenous ligands could also be drugs of clear interest. However, clinical studies with these modulators are still lacking and such studies are critical to demonstrate the efficacy of such molecules in controlling certain pain disorders. While from the above discussion the clinical value of modulation of the first thermoTRP member TRPV1 as a target in some pain settings is clear, other thermoTRP members have also drawn recent attention. 

## TRPV2

Residual noxious heat sensation at temperatures above 52^o^C in TRPV1 knockout mice led to the discovery of the second thermoTRP, originally known as vanilloid receptor like protein 1 (VRL-1) and now renamed TRPV2 [22, 140]. Since its cloning TRPV2 has emerged as an ion channel with distribution and functions not only in nociceptors but also in other tissues.

### Expression Physiology and Pathology 

TRPV2 is localized in medium to large diameter DRG, Trigeminal ganglia and Nodose ganglia neurons representing the Aδ and Aβ nociceptors. TRPV2 distribution in spinal cord include Lissauer's tract and laminae I, II, III and IV of the DH, dorsal column nuclei, posterior column, ventral horn of sections at the lumbosacral junction, ventral horn motoneurons, intermediolateral (IML) cell column composed of sympathetic preganglionic neurons, ependymal cells lining the central canal and astrocytes [3, 22, 87, 115, 241]. Central projections of Aδ nociceptors with TRPV2 in laminae I and II may be involved in nociception, although direct *in vivo* evidence is still lacking. However, it is known that TRPV2 expression in trkC subpopulations of adult DRG’s is dependent on NT-3 signaling in development stages [211]. Since NT-3 is reported to induce mechanical and thermal hyperalgesia followed by mechanical hypoalgesia [126, 184], it is suggested that TRPV2 may play a role in NT-3 mediated thermal hyperalgesia. TRPV2 may also serve non-nociceptive functions. Laminae III and IV, dorsal column nuclei and posterior column, receive large diameter mechano-Aβ sensory fibers involved in proprioception. TRPV2 in the lumbosacral junction may have a functional role towards the urethral sphincter and ischiocavernosus muscles that are innervated by neurons of the dorsolateral nucleus [131, 180]. A role of TRPV2 in CSF transport of molecules is speculated due to its presence in the central canal ependymal cells. The presence of TRPV2 in NG (vagal afferents) and intrinsic neurons of myentric plexus suggest a role for receiving sensory signals from viscera and intestine [86, 100]. Among the viscera, laryngeal innervation is TRPV2 positive and hence suggests a possible role in laryngeal nociception [159]. In the brain, TRPV2 is localized to hypothalamic paraventricular, suprachiasmatic, supraoptic nuclei, oxytocinergic and vasopressinergic neurons and cerebral cortex [116]. Since these areas of the brain have neurohypophysial function and regulation of neuropeptide release in response to changes in osmolarity, temperature, and synaptic input, TRPV2 may have a role in disorders of the hypothalamic-pituitary-adrenal axis, such as anxiety, depression, hypertension, and preterm labor [226]. In a model of peripheral axotomy, TRPV2 was up-regulated in postganglionic neurons in lumbar sympathetic ganglia but not in the DRG, spinal cord or brainstem, suggesting a role in sympathetically mediated neuropathic pain [65]. 

The non-neuronal distribution of TRPV2 includes vascular and cardiac myocytes [90, 144, 160] and mast cells [197]. TRPV2 is activated by membrane stretch, a property relevant for its sensory role in the gut. TRPV2 in cardiac musclemay be involved in the pathogenesis of dystrophic cardiomyopathy [89] and in mast cells, and may play a role in urticaria due to physical stimuli (thermal, osmotic and mechanical). Activation by physical stimuli is discussed in the next section. A functional role for TRPV2 recently found in human peripheral blood cells needs further study [178]. 

### Activation and Regulation

TRPV2 is activated *in vitro* by physical stimuli such as heat, osmotic and mechanical stretch [22, 90, 144] and chemical stimulus by 2-aminoethoxydiphenyborate (2-APB) [80]. Translocation of TRPV2 from intracellular locations to plasma membrane required for its activation is regulated by insulin-like growth factor-I (IGF-I) [99]; A-kinase anchoring proteins (AKAP)/cAMP/protein kinase A (PKA) mediated phosphorylation [197]; G-protein coupled receptor ligands like neuropeptide head activator (HA) *via* phosphatidylinositol 3-kinase (PI3-K) andof the Ca^2+^/calmodulin-dependent kinase (CAMK) signaling [17]. These regulatory mechanisms that induce membrane localization of TRPV2 seem to be important regulation mechanisms for TRPV2 activation.

### Therapeutic Potential

Given the distribution pattern of TRPV2 in sensory afferents and their projections, the predicted physiological and pathological role in mediating pain makes it an important target for certain pain states in addition to TRPV1. However, progress into TRPV2 pharmacology, unlike TRPV1 has been patchy and requires more investigations to determine its niche in pain biology. *In vivo* evidence for thermal and mechanical nociception *via* TRPV2 is still elusive. 2-APB, the only known chemical activator of TRPV2, is non-selective. Ruthenium Red (RR) a general blocker of TRPV ion channels is non-selective antagonist of TRPV2. The lack of specific tools and knockout animal models has impeded detailed investigations into TRPV2 function in physiology and pathology. Future efforts in this direction are awaited.

## TRPA1

The ankyrin-repeat transient receptor potential (TRPA) channel subfamily has currently a single member named TRPA1 (previously coined p120, ANKTM1 or TRPN1), with characteristic long ankyrin repeats in its N-terminus [92, 94,139,199]. A role for TRPA1 in somatosensation is currently not without inconsistencies due to variable pain assay methods. Evidence for TRPA1 as a thermoTRP directly activated by noxious cold [11, 199] could not be reproduced by later studies using *in vivo* TRPA1 knockout model or other heterologous expression systems [12, 94]. However, another independent knockout study showed a cold response role for TRPA1 [112]. Nevertheless, sensory transduction of cold-induced pain by TRPA1 seems to draw attention. Evidence for distribution and function in nociceptors makes TRPA1 an exciting new therapeutic target to achieve analgesia.

### Expression, Physiology and Pathology

TRPA1 and TRPV1 are co-expressed in C and Aδ nociceptors from DRG, nodose ganglia and trigeminal ganglia [105,145, 199], making these transducers of both noxious cold and heat-induced pain. TRPA1 is also expressed in sympathetic neurons from the superior cervical ganglion [191] and neurons of the geniculate ganglia [102], suggesting a role in oral sensory transduction. Non-neuronal expression of TRPA1 is currently limited to lung fibroblasts (as ANKTM1) [92] and hair cell stereocilia [36, 145] where it may serve as a mechanotransducer. Other non-neuronal expression was found at mRNA levels in small intestine, colon, skeletal muscle, heart, brain, and immune system. Nociceptive afferents expressing TRPA1 innervate bladder [8], suggesting a role in bladder contraction. Upregulation of TRPA1 expression is observed in pathological pain models like cold hyperalgesia induced by inflammation and nerve damage [155]; exaggerated response to cold in uninjured nerves during spinal nerve ligation [101]; cold allodynia during spinal nerve injury [7]; bradykinin (BK)-induced mechanical hyperalgesia and mechanical pin prick pain [11, 112]. Due to normal auditory behaviour in TRPA1 knock out studies, its role in hearing has been ruled out [12, 112], and hence its role in hair cell mechanotransduction remains challenged [36]. Further studies are necessary to clearly define pain mechanisms mediated *via* TRPA1. Also, further evaluation TRPA1 expression and function using knockout studies are required with emphasis on cold- and mechano-transduction mechanisms. 

### Activation and Regulation

Similar to TRPV1, TRPA1 pharmacology has made great strides since the receptor was found to respond to pungent ingredients from natural products. 

#### Isothiocyanates

TRPA1 can be selectively activated by pungent ingredients like allyl, benzyl, phenylethyl, isopropyl, and methyl isothiocyanate, from wasabi, yellow mustard, Brussels sprouts, nasturtium seeds, and capers, respectively [94]. However, its involvement in burning pain induced by the mustard oil derivative allyl isothiocyanate in variable subsets of nociceptors is debated [12, 24, 94, 112]. 

#### Cinnamaldehyde

Cinnamaldehyde, the main pungent constituent from cinnamon oil, activates TRPA1 [11]. Acute burning pain sensation caused by cinnamaldehyde is suggested to be mediated by TRPA1 expressed in nociceptors that project to the tongue and skin [11].

#### Δ^9^-tetrahydrocannabinol

THC, a cannabinoid, activates TRPA1 and is suggested to induce some of its biological effects, like dilation of hepatic or mesenteric arteries *via* activation of capsaicin-sensitive, CGRP-containing perivascular sensory nerve endings innervating the smooth muscle [247]. THC also activates TRPA1 in trigeminal neurons [94]. Hence, cannabinoid mechanisms may play an important role by interacting with the TRPA1 component in these nociceptors.

#### Acrolein

Acrolein (2-propenal), a higly toxic air pollutant in tear gas, vehicle exhaust, and smoke from burning vegetation, including tobacco products [72, 73] selectively activated TRPA1 [12]. Thus biological effects of acrolein, like apnea, shortness of breath, cough, airway obstruction, and mucous secretion [67] may result from TRPA1 activation in TRPV1- and CGRP-positive afferent innervations of airway. Chemotherapeutic agents like cyclophosphamide and ifosfamide for cancer, severe arthritis, multiple sclerosis, and lupus [62, 149] generate acrolein as a metabolite, suggesting that TRPA1 may be involved in the side effects of such conditions. Studies using heterologous expression and knockout systems rule out acrolein as a TRPV1 agonist [47, 204].

#### Fatty Acid Amide Hydrolase (FAAH) Inhibitor

3'-carbamoylbiphenyl-3-yl cyclohexylcarbamate (URB 597), a potent and systemically active inhibitor of FAAH (the enzyme responsible for anandamide degradation) was recently shown to directly gate TRPA1 and is being pursued as an antinociceptive drug [150].

#### Non-Selective Activators

These include eugenol (from clove oil), gingerol (from ginger), and methyl salicylate (from Wintergreen oil), synthetic AG-3-5 (Icilin) [132, 200], all of which non-selectively activate TRPV1 and TRPM8. Allicin, thought to be a non-selective activator of TRPV1 and TRPA1 [123] is now being considered as a selective agonist for TRPA1 [12].

#### Modulators

Like TRPV1, hypersensitivity of TRPA1 is coupled to G-protein mediated BK signaling and contributes to mechano- and cold-hyperalgesia [11, 112]. Noguchi and colleagues showed that an increase in NGF-induced TRPA1 in nociceptors *via* p38 MAPK activation was necessary for cold hyperalgesia [134, 155]. TRPA1 is potentiated by extracellular signal-regulated protein kinase (ERK) and PLC disinhibition of PIP2 *via* proteinase activated receptor (PAR)-2 mediated activation in models of thermal hyperalgesia and inflammatory pain [42, 103, 135]. These studies provide further insights into TRPA1 signaling. Like the TRPV1, PLC-medi-ated pathway sensitization of TRPA1 has been shown [132]. Activation of Mu and Kappa opioid receptors antagonized the stimulant action of icillin on TRPA1 [232], suggesting a central mechanism of interaction between opioid receptors and TRPA1. Evidence for TRPA1 as a substrate for ubiquitination by CYLD (an ubiquitin hydrolase and a tumor suppressor gene product) along with wide tissue distribution indicates a probable role in cancer [198]. Further studies are necessary to identify wider functional TRPA1 protein expression. Evidence for indirect gating of TRPA1 by cold is shown to be regulated by calcium binding domain (EF hand) in the N-terminus [50, 245]. Artemin, a glial cell line-derived neurotrophic factor (GDNF) protein, was shown to increase TRPA1 gene expression in skin and is suggested to mediate cold allodynia during inflammation [57]. Most of these signaling mechanisms involving TRPA1 sensitization of pain states need to be addressed using TRPA1 knockout studies in tandem with TRPV1 knockout models.

### Therapeutic Potential

Evidence for TRPA1 as a transducer of pain is certainly on the rise, making it yet another important target for therapy. The therapeutic potential of TRPA1 for appropriate pharmacological treatment of certain pain states needs further investigation. Unlike TRPV1, the agonists of TRPA1 currently are only known to produce pain and hence antagonists are a better choice than agonists as analgesics. One recent published work describes identification of potential TRPA1 anatagonists using a novel transient expression system screening method [27]. Development of these substances is an important step for elucidating the role played by TRPA1 in painful conditions. Since activation of TRPA1 in nociceptors induces pain behaviour, design of specific antagonists seems beneficial. Since other physiological roles of TRPA1 are under debate, further research into its pharmacology would help in choosing agonists versus antagonist drugs.

## TRPM8

TRPM8 (Trp-p8 or CMR1), is a channel belonging to the TRPM (long or melastatin) subfamily of TRP channels, with a characteristic lack of ankyrin repeat domains in the N-terminus [34, 130, 140, 165, 217]. The channel was cloned initially as an upregulated protein in prostate [217]. Later it was discovered as a thermoTRP for cool and menthol sensation by two groups- one used an expression screening strategy (similar to TRPV1 cloning) for a menthol- and cold-sensitive receptor [130], while the other used genomic DNA databases for TRP protein sequences [165]. The threshold for TRPM8 activation is about 25 °C, a temperature in the non-noxious range. Long awaited studies on the role of TRPM8 in nociceptors using knockout strategies have now been published [13, 35, 46]. These studies have shown that TRPM8 can serve as thermosensor for cold and mediate both cold-induced nociception as well as analgesia. However, the TRPM8 knockout mice retained response to intense cold temperatures below 10 ^o^C, indicating the presence of other thermosensors. A study involving mice with double knockout of TRPA1 and TRPM8 would perhaps eliminate the entire range of cool to cold temperature sensation. However, this remains to be seen as, Koltzenburg and colleagues have shown the presence of a third population of cold-sensitive neurons distinct from the TRPA1 and TRPM8 population [143].

### Expression Physiology and Pathology

Interestingly, TRPM8 is expressed in a subset of sensory neurons of C and Aδ class in DRG, trigeminal ganglia and nodose ganglia that are negative for nociceptor markers TRPV1, CGRP and IB4 [130, 147, 165, 172]. A recent strategy to generate transgenic mice with GFP under the control of TRPM8 promotor has good potential to study distribution and function in its physiology and pathology [210]. Neuronal expression and knockout studies implicate TRPM8 for a somatosensory role in cool temperature sensation [13, 35, 46, 130, 165]. It is believed that TRPM8 activation leads to analgesia during neuropathic pain. Evidence for such an analgesic mechanism was recently shown to be centrally mediated, whereby TRPM8-induced glutamate release activates inhibitory Group II/III metabotropic glutamate receptors (mGluRs) to block nociceptive inputs [168]. However, a role for TRPM8 in innocuous cold nociception has also been shown [69, 227]. The TRPM8 knockout mice studies more clearly point towards a role for TRPM8 in sensory neurons in physiological (somatosensation) and pathological conditions (cold pain), especially owing to their presence in C and A( fibers, generally regarded as nociceptors [13, 35, 46]. 

The non-neuronal expression of TRPM8 is currently restricted to prostate, urogenital tract, taste papillae, testis, scrotal skin, bladderurothelium, thymus, breast, ileum and inmelanoma, colorectal cancer and breast cancer cells [1, 195, 217, 240, 241]. The physiology of TRPM8 in non-neuronal tissues is well described elsewhere [240]. 

### Activation and Regulation

TRPM8 pharmacology has also progressed considerably due to availability of a number of agonists and antagonists. Several studies have also been conducted to understand regulatory mechanisms of the receptor. 

#### Terpenes

Menthol, derived from peppermint oil, cornmint oil, citronella oil, eucalyptus oil, and Indian turpentine oil, activates TRPM8 in sensory neurons of DRG and TG [130, 165]. Menthol sensitizes TRPM8 to cold stimulus [172]. However, menthol is now known to non-selectively activate and sensitize TRPV3 [124]. Eucalyptol derived from *Eucalyptus polybractea* activates TRPM8 with lower efficacy than menthol. It is used in as an analgesic for inflammatory and muscular pain [20].

Menthone, geraniol, linalool, menthyl lactate, *trans*- and *cis*-*p*-menthane-3,8-diol, isopulegol, and hydroxy-citronellal are other terpene compounds known to activate TRPM8 [11, 14] by mechanisms that need further analysis. 

#### Non-Terpenes

Icilin (AG-3–5), WS23, WS3, Frescolat ML, Frescolat MGA, and Cooling-agent 10 are some of the non-terpene compounds that have been shown to effectively activate and desensitize TRPM8 [20].

#### Antagonists

Non-selective antagonists of TRPM8 include capsazepine, *N*-(4-tert. butyl-phenyl)-4-(3-chloropyridin-2-yl) tetrahydropyrazine-1 (2H)-carboxamide (BCTC) and a thio-derivative of BCTC, (2*R*)-4-(3-chloro-2pyridinyl)-2-methyl-*N*-[4-(tri-fluoromethyl)phenyl]-1 piperazonecarboxamide(CTPC) and SB-452533 [14, 231]. Surprisingly, 2-APB, an activator of TRPV1, 2 and 3 is an antagonist of TRPM8 [80]. 2-APB could be useful in characterizing TRPM8 mechanisms selectively. Agonists of TRPA1 like cinnamaldehyde and URB597 are shown to antagonize TRPM8 [124, 150].

#### Modulators

Voltage dependence of TRPM8 during cold and menthol activation suggests its dependence on membrane potential for activation [19, 84, 213].PIP_2_ was shown to be essential for activation of TRPM8, and PIP2 depletion *via* PLC pathway activation resulted in desensitization [15, 119, 174]. Activation of TRPM8 by icilin was shown to be dependent on intracellular calcium [29]. Calcium-independent and iPLA2-dependent activation of prostate TRPM8 by lysophospholipids (metabolites of iPLA2) provides a first evidence for endogenous ligands in non-neuronal tissue not exposed to cooling [220]. This mechanism has not been attributed to sensory transduction by TRPM8. A structural component necessary for formation and trafficking of functional TRPM8 to plasma membrane lies in the coiled-coil C-terminal region [58]. Other structural motifs necessary for channel activation are two cysteine residues in the pore region flanked by the glycosylation site [54]. Such studies are useful to understand the channel function in response to specific modalities, where TRPM8, like other thermoTRP’s, is polymodal. 

Since TRPM8 activation can mediate both pain and analgesia, it is necessary to develop both agonists and antagonists, as seen in the case of TRPV1 for pain management. 

### Therapeutic Potential

As is the case of TRPA1, therapeutic potential of TRPM8 with existing data makes it a target to achieve analgesia during cold pain. Unlike TRPA1, either activation or blockade of TRPM8 is therapeutically useful depending on the modalities of different pain settings. TRPM8 can also be an important target for identification and or therapy of cancer in prostate, breast, colon, lung and skin.

## TRPV3

TRPV3 is the other thermoTRP that responds to innocuous temperatures with a threshold higher than TRPV4 [166, 190]. Expression of TRPV3 among sensory neurons is variable between species and thus its role in somatosensation needs further investigations [166, 190, 239]. However, an increase in TRPV3 expression in peripheral nerves after injury and in avulsed DRG is documented [60]. Evidence for a role of TRPV3 in thermosensation has emerged with demonstration of its presence in the keratinocytes [31, 32, 166, 239] and aberrant thermal selectivity in TRPV3 knockout study [141]. In addition, gene knock out studies have shown hair loss [10]. CNS expression of TRPV3 includes ventral motor neurons, deeper laminae of DH, superior cervical ganglion neurons, nigral dopaminergic neurons [70, 60, 190, 239]. A physiological role for TRPV3 in these areas needs further investigation. A functional role for TRPV3 in pain is not yet well established. Some studies may point towards this direction. One study showed an increase in TRPV3 expression following brachial plexus avulsion, however, its symptoms are not pain related [190]. Another feature of TRPV3 which prompts its possible role in pain is its sensitization upon repeated heat applications in skin cells and heterologous expression systems, a phenomenon yet to be confirmed in sensory neurons [32, 141, 166]. An increase in expression was also seen in skin cells during breast pain in addition to TRPV1 upregulation in nociceptors [68]. A recent study showed that TRPV3 in oral and nasal epithelium is activated and sensitized by non-selective pungent compounds like carvacrol, thymol, eugenol, 6-tert-butyl-m-cresol, dihydrocarveol, carveol and (+)-borneol [222, 237]. Camphor, menthol, 2-APB and diphenylboronic anhydride (DPBA) are the other non-selective agonists of TRPV3 which can activate and sensitize it to repeated applications [33, 124]. Also TRPV3 response to heat is sensitized in the presence or prior applications of its chemical agonists [141, 237]. Since camphor and menthol exhibit opposite thermal properties of warm and cool sensation, a role for TRPV3 in coding for cool sensation is not proven. Such paradoxes need to be resolved. TRPV3 pharmacology will also need more than the existing two known non-selective antagonists of TRPV3, ruthenium red and diphenyltetrahydrofuran (DPTHF) [33]. Chemical stimulation of TRPV3 by these non-selective compounds increases the complexity of TRPV3 pharmacology and more detailed characterization and physiological role in pain models need to be addressed before it can be considered a validated target for such therapy. Interestingly, a recent study has shown that TRPV3 can be potentiated by arachidonic acid and its metabolites, the endogenous mediators of inflammatory response in skin cells [81]. The close proximity of nociceptors to skin epidermal layers, where functional TRPV3 is present, makes it a potential molecule with an extended role in physiology and pathology of thermosensation and pain [26, 114]. Such an extended role may involve activation of TRPV3 in the skin followed by release of some diffusible neuroactive substances like ATP that interact with the terminals of the nociceptors. The importance or contribution of TRPV3 in disease and therapy require further investigations. Development of antagonists for TRPV3 seems beneficial to overcome its sensitization in pain settings. However, these agents may have side effects of skins barrier integrity.

## TRPV4

Innocuous warm temperatures (27-32^o^C) activate the thermoTRP TRPV4 (previously coined with several names, including VRL-2, OTRPC4, VR-OAC and TRP12). It was first identified as an osmoreceptor [71, 117, 201, 228, 235]. TRPV4 is reported to have a wide tissue distribution pattern and to function in both excitable and non-excitable cells. TRPV4 knockout studies have resulted in an intensive examination of function in physiology and pathology. Here we focus on pain mechanisms that have emerged recently.

### Expression, Physiology, Pathology

Expression of TRPV4 in the nociceptor class of sensory neurons in DRG and trigeminal ganglia earlier limited to mRNA studies [71, 117] is now extended to functional protein expression. TRPV4 is involved in mechanical hyperalgesia and allodynia following its sensitization during hypo-osmotic mediated inflammation, taxol-induced neuropathy or mild hypertonic insult [4-6]. These studies have shown that PGE2 and PAR2 mediated sensitization of TRPV4 occurs during inflammatory hyperalgesia. TRPV4 knockdown studies have also shown impaired sensitivity to acid, increase in mechanical nociceptive threshold with unchanged response to heat and touch and reduced thermal hyperalgesia [118, 202, 214]. However, more studies are necessary to confirm if there is a central component to the diminished mechanical pain behavioral phenotype observed in TRPV4 knockout studies. The CNS expression includes neurons of circumventricular organs, ependymal cells of choroids plexus, cerebral cortex, thalamus, hippocampus, and cerebellum [117]. A role for TRPV4 in regulating excitability of mouse hippocampal neurons at physiological body temperature has recently been demonstrated [182].

Numerous studies provide evidence for TRPV4 as being a crucial mechano- or osmo-receptor in other cell types, such as vascular aortic endothelial cells, blood–brain barrier endothelial cells, renal collecting duct cells, vascular smooth muscle cells, hypothalamus (neurons of the circumventricular organs and the organum vasculosum of the lamina terminalis with projections to the magnocellular regions of the supraoptic and paraventricular nuclei) and cochlear hair cells [161]. Expression of TRPV4 in keratinocytes and its response to warm temperatures has raised the possibility of a sensory role of thermoTRP’s in non-neuronal cells [31, 32, 71]. Aberrant thermal selection in TRPV4 knockout studies provided physiological evidence for its role in thermosensation [114]. 

### Activation and Regulation

In addition to physical stimuli like heat, pressure and hypotonicity, chemical activation of TRPV4 include exogenous and endogenous ligands. TRPV4 pharmacology has had mixed progress in light of the non-availability of selective antagonists.

#### Synthetic Phorbol Esters

4α-phorbol 12,13-didecanoate (4α-PDD) and other non-active 4( phorbol ester isomers selectively activate TRPV4 [228, 236] active phorbol esters like PMA, PDD and PDBu are agonists of TRPV4 at warmer temperatures and activate TRPV4 in a PKC dependent manner [236].

#### Endogenous Second Messenger Metabolites

TRPV4 is directly activated by anandamide (AEA) and its LOX metabolite arachidonic acid (AA) [229]. Further, epoxyeicosatrienoic acid (EET) metabolites of AA formed by cytP450 epoxygenase pathway (5,6-EET; 8,9-EET; 11, 12-EET) also activate TRPV4 [223]. Other endogenous activators of TRPV4 include N-acyl taurines (NAT’s), which are fatty acid amides regulated, by fatty acid amide hydrolase (FAAH) [176].

#### Plant Extracts

Like other thermoTRP’s activated by natural compounds, a very recent study has identified a natural compound bisandrographolide A (BAA) contained in extracts of the plant *Andrographis paniculata* to activate TRPV4 [192].

#### Intracellular Components as Modulators

The presence of intracellular components that interact and regulate TRPV4 channel expression and function were evident from the fact that it cannot be activated by heat in a membrane de-limited condition [228], necessitating the presence of intracellular components as modulators. A number of studies in this direction have emerged. Inhibition of 4α PDD-induced TRPV4 activity was inhibited by an increase in both extracellular and intracellular calcium, and this modulation was dependent on amino acid residues in the 6^th^ transmembrane domain (F707), pore region (D682) and C-terminus (E797), whereby increased extracellular calcium has an inhibitory effect on the channel [230]. Phorbolesters and heat activation rely on aromatic residue Tyr-556 at the N terminus of transmembrane domain 3 [224] and two hydrophobic residues Leu-584 and Trp-586 in the central part of transmembrane domain 4 [225]. However, in addition to phorbol esters and heat, responses to cell swelling, arachidonic acid, and 5,6-EET were affected by mutations of two residues Tyr-591 and Arg-594 in the C-terminal part of transmembrane domain 4 [225]. These residues of transmembrane domains 3 and 4 are thus essential for channel gating and ligand binding affinity for TRPV4 [224, 225]. Lyn, a member of Src-family of tyrosine kinases, mediated tyrosine phosphorylation at Tyr-253 residue to regulate TRPV4 response to hypotonic stress [224, 236]. Glycosylation of TRPV4 at N651 residue of the pore loop region results in inhibition of membrane trafficking and thus a decreased channel response to hypotonicity [238]. Association of aquaporin 5 (AQP5) with TRPV4 initiates a regulatory volume decrease (RVD) mechanism following hypotonic stimulus in epithelial cells [122, 186]. PACSINs, the regulators of synaptic vesicular membranetrafficking and dynamin-mediated endocytotic processes, were shown to interact with the amino terminus of TRPV4 and increase plasmamembrane-associated TRPV4 protein. The interaction was found between TRPV4-specific proline-richdomain upstream of the ankyrin repeats of the channel and thecarboxyl-terminal Src homology 3 domain of PACSIN 3 [39]. A cytoskeletal protein, microfilament-associated protein (MAP7), was shown to interact with TRPV4 and form a mechanosensitive molecular complex to drive and enhance membrane expression of the ion channel [203]. MAP7 interacts with the C-terminus domain between amino acid residues 789-809. The serine/threoninekinases "With No Lysine (K) Kinases" (WNK)1 and WNK4 were also shown to interact with TRPV4 and reduce its cell surface expression, inhibiting response to activators like 4αPDD and hypotonicity [63]. 

The list of intracellular components that interact with TRPV4 may increase in future due to its wide distribution and function in various tissues. This will help understand the regulatory events controlling TRPV4 in health and disease.

### Therapeutic Potential

As pointed out earlier in this section of the review, so far there is no report of specific antagonists to enhance the detailed pharmacological characterization in pain conditions involving TRPV4. However, such developments are necessary to achieve novel therapeutic strategies for TRPV4-dependent pain states. TRPV4 knockout studies have however revealed a physiological role and predicted involvement of TRPV4 in diseases like thermal hyperalgesia, neuropathic pain, hyperresponsive airway during asthma, hypotonic stress during cystic fibrosis, impairment of hearing [152]. In parallel to TRPV1, more studies are necessary to elucidate sensitization mechanisms of TRPV4 in pain settings when exposed to physical stimuli like heat, hypotonic and mechanical stress. Given the wide distribution, function and complexity in modulation of TRPV4, it would be a challenge to target the receptor with agonists or antagonists for specific disease states.

## CONCLUSION

ThermoTRPs in nociceptors have emerged as potential targets for the treatment of pain with a broader perspective. Much progress has been made in the last decade since the cloning of TRPV1. Clinical trials for TRPV1 as a target for selected pain modalities is clear evidence for continuing efforts to search for other thermoTRP transducers of pain in nociceptors. Much effort is needed to establish the role of thermoTRP’s as potential targets in pain and other therapy and the progress so far has been quite rapid. TRPV1 studies definitely lead the way to providing the basis for the search for remaining thermal transducers of pain in nociceptors. Future work on TRPV1 and other thermoTRP’s will enhance our understanding of somatosensation in health and disease. Availability of animal models especially knockout mice of each of the above mentioned thermoTRP’s are vital to rapidly advance thermoTRP research. Much effort is warranted towards development of more specific drugs (agonists/anta-gonists) to target each thermoTRP for progress in understanding TRP pharmacology.

## Figures and Tables

**Table 1.** Distribution of TRPV1, TRPV2, TRPA1, TRPM8, TRPV3 and TRPV4

ThermoTRP	Neuronal Distribution	Non-Neuronal Distribution
TRPV1	dorsal root ganglia; trigeminal ganglia; jugular ganglia; nodose ganglia; solitary tract nucleus; trigeminal nucleus; ventral medulla; periaqueductyl grey; dorsal raphe nucleus; locus coeruleus; hypothalamus; thalamus; hippocampus; ventral tegmental area; cerebel-lum; substantia nigra; somatosensory cortex	epithelial cells of the GI, airway and bladder; epidermal keratinocytes from human skin; enterocytes; liver; vascular endothelium; mast cells; smooth muscle; fibroblasts; peripheral mononuclear blood cells.
TRPV2	dorsal root ganglia; trigeminal ganglia; nodose ganglia; spinal cord Lissauer's tract, dorsal column nuclei, posterior column, ventral horn, motoneurons, sympathetic preganglionic neurons, central canal ependymal; hypothalamic paraventricular nuclei, suprachiasmatic nuclei, supraoptic nuclei, oxytocinergic and vasopressinergic neurons; cerebral cortex	vascular and cardiac myocytes; mast cells; astrocytes; spleen; lung; intestine; vas deferens
TRPA1	dorsal root ganglia; nodose ganglia; trigeminal ganglia; superior cervical ganglion; geniculate ganglia	lung fibroblasts; hair cell stereocilia; intestine; skeletal muscle; heart; immune system
TRPM8	dorsal root ganglia; trigeminal ganglia; nodose ganglia	prostate; urogenital tract; taste papillae; testis; scrotal skin; bladder urothelium; thymus; breast; ileum;
TRPV3	dorsal root ganglia; motor neurons; superior cervical ganglia; nigral dopaminergic neurons	keratinocytes; hair follicle sheath cells; skeletal muscle; pituitary; intestine
TRPV4	dorsal rrot ganglia; trigeminal ganglia; circumventricular organs; choroids plexus; cerebral cortex; thalamus; hippocampus; cerebellum; hypothalamus	vascular aortic endothelium; blood–brain barrier endothelium; renal collecting duct; vascular smooth muscle; cochlea; keratinocytes

**Table 2.** Ligands for TRPV1, TRPV2, TRPA1, TRPM8, TRPV3 and TRPV4

ThermoTRP	Non-endogenous	Endogenous
TRPV1	vanilloids (capsaicin, resiniferatoxin, piperine, eugenol, gingerols, capsiate, NGX-4010, WL-1002); non-vanilloids (ginsenosides, cannabidiol, evodia compounds, unsaturated 1,4-dialdehyde terpenes, triprenyl phenol, polygodial and drimanial, unsaturated 1,4-dialdehyde sesquiterpenes, 2-APB, camphor)	N-arachidonoyldopamine (NADA); oleoylethanolamide (OEA); N-oleoyldopamine (NODA); anandamide; 12- and 15-(S)-hydroperoxyeicosatetraenoic acids (12- and 15-HPETE), 5- and 15-(S)-hydroxyeicosatetraenoic acids (5- and 15-HETE); leukotriene B4; polyamines (spermine)
TRPV2	2-APB	
TRPA1	isothiocyanates; cinnamaldehyde; THC; acrolein; eugenol; methyl salicylate; icilin; gingerol; URB597	calcium
TRPM8	terpenes (menthol, eucalyptol, Menthone, geraniol, linalool, menthyl lactate, trans- and cis-p-menthane-3,8-diol, isopulegol, hydroxy-citronellal); non-terpenes (Icilin (AG-3–5), WS23, WS3, Frescolat ML, Frescolat MGA, Cooling-agent 10)	lysophospholipids
TRPV3	carvacrol; thymol; eugenol; 6-tert-butyl-m-cresol; dihydrocarveol; carveol; (+)-borneol; camphor; menthol; 2-APB; diphenylboronic anhydride (DPBA); cinnamaldehyde	
TRPV4	synthetic phorbol esters; bisandrographolide A (BAA)	anandamide (AEA); arachidonic acid (AA); epoxyeicosatrienoic acid (EET) metabolites (5,6-EET; 8,9-EET; 11,12-EET); N-acyl taurines (NAT’s)

**Table 3.** Cell Signaling Modulators of TRPV1, TRPV2, TRPA1, TRPM8, TRPV3 and TRPV4

ThermoTRP	+ve Modulators	-ve Modulators
TRPV1	PKC, PKA, CAMKII, ERK, PI3K	calcineurin, calmodulin; PIP2
TRPV2	IGF-I; AKAP/cAMP/PKA; PI3K; CAMK	
TRPA1	p38 MAPK; ERK; PLC; Artemin	pyrophosphate (PPi); polytriphosphate (PPPi)
TRPM8	PIP2; calcium	PKC; PKA
TRPV3	arachidonic acid and metabolites; PKC	
TRPV4	Src-tyrosine kinase; AQP5; PACSINs; MAP7	WNK1; WNK4

**Table 4.** Antagonists for TRPV1, TRPV2, TRPA1, TRPM8, TRPV3 and TRPV4

ThermoTRP	Antagonists
TRPV1	capsazepine; ruthenium red; diphenyltetrahydrofuran (DPTHF); iodo-RTX; SB705498; SB366791; BCTC; NGD-8243; AMG-517; AMG-9810; A-425619; KJM429; JYL1421; JNJ17203212; NGX-4010; WL-1001; WL-1002; A-4975; GRC-6127; 2-(4-pyridin-2-ylpiperazin-1-yl)-1H-benzo[d]imidazole compound 46ad; 6-aryl-7-isopropylquinazolinones; 5,6-fused heteroaromatic urea A-425619.0; 4-aminoquinazoline; halogenated thiourea compounds 23c and 31b; N-tetrahydroquinolinyl, N-quinolinyl and N-isoquinolinyl carboxamides; pentacyclic triterpene; oleanolic acid;
TRPV2	ruthenium red; diphenyltetrahydrofuran (DPTHF)
TRPA1	ruthenium red; camphor; menthol; compoud A and compound B (Abbott Laboratories)
TRPM8	capsazepine; BCTC; CTPC; SB-452533; 2-APB; URB597; cinnamaldehyde
TRPV3	ruthenium red; diphenyltetrahydrofuran (DPTHF)
TRPV4	ruthenium red
